# Stability of radiomics features in apparent diffusion coefficient maps from a multi-centre test-retest trial

**DOI:** 10.1038/s41598-019-41344-5

**Published:** 2019-03-18

**Authors:** Jurgen Peerlings, Henry C. Woodruff, Jessica M. Winfield, Abdalla Ibrahim, Bernard E. Van Beers, Arend Heerschap, Alan Jackson, Joachim E. Wildberger, Felix M. Mottaghy, Nandita M. DeSouza, Philippe Lambin

**Affiliations:** 1 The D-Lab, Department of Precision Medicine, GROW - School for Oncology and Developmental Biology, Maastricht University Medical Centre+, Maastricht, The Netherlands; 2 Department of Radiology and Nuclear Medicine, GROW - School for Oncology and Developmental Biology, Maastricht University Medical Centre+, Maastricht, The Netherlands; 3 Cancer Research UK Cancer Imaging Centre, The Institute of Cancer Research and Royal Marsden Hospital, Sutton, UK; 4 Laboratory of Imaging Biomarkers, UMR 1149 Inserm - University Paris Diderot, Paris; Department of Radiology, Beaujon University Hospital Paris Nord, Clichy, France; 5 Department of Radiology, Radboud University Nijmegen Medical Center, Nijmegen, The Netherlands; 6 Wolfson Imaging Centre, Wolfson Molecular Imaging Centre, University of Manchester, 23 Palatine Rd, Withington, Greater Manchester UK; 7 Department of Nuclear Medicine, University Hospital RWTH Aachen University, Aachen, Germany

## Abstract

Quantitative radiomics features, extracted from medical images, characterize tumour-phenotypes and have been shown to provide prognostic value in predicting clinical outcomes. Stability of radiomics features extracted from apparent diffusion coefficient (ADC)-maps is essential for reliable correlation with the underlying pathology and its clinical applications. Within a multicentre, multi-vendor trial we established a method to analyse radiomics features from ADC-maps of ovarian (n = 12), lung (n = 19), and colorectal liver metastasis (n = 30) cancer patients who underwent repeated (<7 days) diffusion-weighted imaging at 1.5 T and 3 T. From these ADC-maps, 1322 features describing tumour shape, texture and intensity were retrospectively extracted and stable features were selected using the concordance correlation coefficient (CCC > 0.85). Although some features were tissue- and/or respiratory motion-specific, 122 features were stable for all tumour-entities. A large proportion of features were stable across different vendors and field strengths. By extracting stable phenotypic features, fitting-dimensionality is reduced and reliable prognostic models can be created, paving the way for clinical implementation of ADC-based radiomics.

## Introduction

Diffusion-weighted magnetic resonance imaging (DWI) is widely used in oncology for identification and characterization of tumours^1^, as well as localization^2^. Signal attenuation in DWI arises from Brownian motion of water molecules and reflects their interaction with cellular barriers and tissue macromolecules that restrict their mobility. DWI is used for tumour characterization and as an indirect biomarker of tissue cellularity^3^. By incorporating a number of diffusion-sensitizing gradients with varying strength, duration and time interval (i.e., b-values) into the MR pulse sequence, parametric apparent diffusion coefficients (ADC) maps can be derived. In oncology, ADC maps are used to determine tumour malignancy and assess early treatment response by quantifying the diffusion-related attenuation of MR signal intensity^3,4^. There is no consensus regarding the threshold value below which ADC is indicative of tumour, however ADC values around 1000 × 10^−6^ mm^2^/s are considered normal, while lower values generally reflect restricted diffusion that could relate to hyper-cellularity or hyper-viscosity characteristic of tumour tissue, and higher ADC values represent fluid filled regions where water diffusion is unrestricted (e.g., cystic lesions, necrotic tissue). Unfortunately, an overlap between ADC values characteristic of active and treated tumours often reduces the utility of ADC in clinical decision-making and variations in estimates of ADC resulting from lack of standardized DWI protocols do not allow the integration of absolute values of ADC as an objective, quantifiable biomarker for personalised healthcare in the clinic^5–7^.

Radiomics may provide complementary information from ADC maps by high-throughput extraction of quantitative tumour phenotypic features (i.e., shape, texture, signal intensity, and wavelet features) that have previously been correlated with tissue pathology and treatment response prediction^8,9^. Please view https://youtu.be/Tq980GEVP0Y for more information. This methodology has shown promising results for CT imaging in oncology and in the development of prediction modelling for treatment response and outcome^10,11^. For DWI, the variability of ADC across different MR systems, vendors and magnetic field-strengths potentially compromises the stability of radiomics features, limiting the clinical implementation of MR radiomics. In addition, there is a realistic risk of overfitting when building a model where the numbers of features extracted greatly outnumber the size of the study cohort. The majority of published radiomics analyses remove features due to redundancy, either because features correlate highly with each other, or because they do not correlate with the endpoint being analysed. Reducing the number of unstable features substantially increases the reliability of radiomics analyses and may not only improve correlation with underlying pathology and tumour biology, but may also allow construction of improved prognostic or predictive models^12–14^. Elimination of features with poor clinical reproducibility is critical for achieving a high radiomics quality score^15^.

The purpose of this analysis was to evaluate the stability of radiomics features extracted from ADC maps, derived from standardized test-retest DWI acquisitions embedded in prospective multicentre trials. DWI for each subject were acquired twice within 7 days, under similar conditions. We hypothesized that selected ADC-based features are generalizable and unaffected by different sources of data variability (i.e., tumour type, MR system, magnetic field strength) when applying standardized protocols on quality-assured MR scanners across multiple clinical centres. Establishing this methodology and presenting the results derived from the underpinning study would not only stimulate the clinical implementation of MR radiomics using ADC as a biomarker for tumour phenotyping^16^ but would also outline a generalizable method for other quantifiable MRI parameters.

## Results

### Feature stability

**Figure 1:**
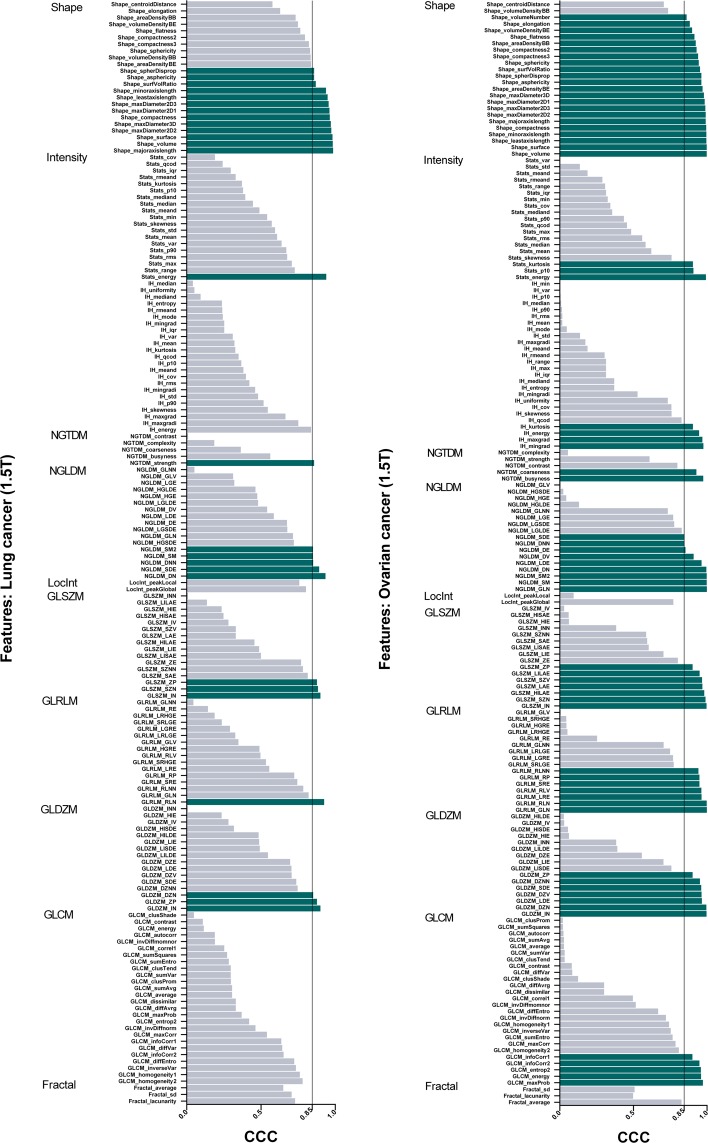
Stability of test-retest radiomics features for all lung and ovarian. cancers The threshold for stability was set at concordance correlation coefficients (CCC) greater than 0.85.

**Figure 2:**
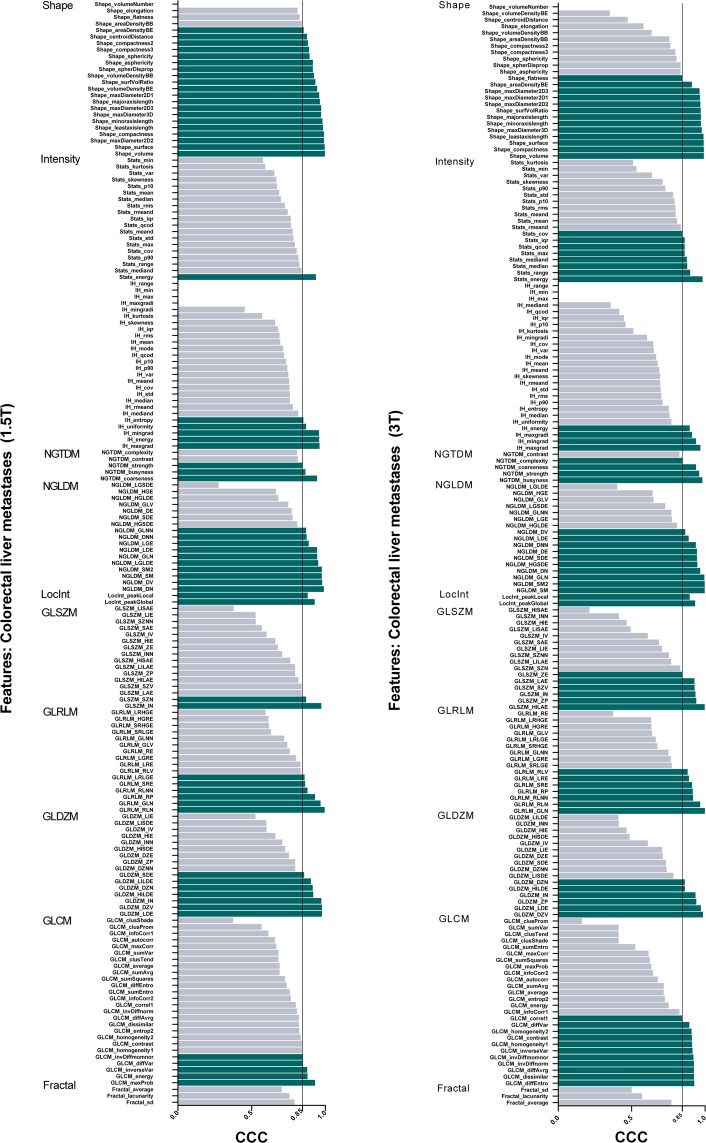
Stability of test-retest radiomics features for all collorectal liver metastases acquired at 1.5 T and 3 T. The threshold for stability was set at concordance correlation coefficients (CCC) greater than 0.85.

Based on previous work, we selected a threshold of 0.85 for the concordance correlation coefficient (CCC), whereby features above this threshold were considered stable between test and retest scans^17^. Figures 1 and 2 show an overview of the stability of test-retest radiomics features for all patient cohorts.

#### Tumour-type differences at 1.5 T

In 20 ovarian cancer lesions from 12 patients, 29% of all features (378/1322) were stable in test-retest ADC maps (Table 1). Of the unfiltered features, most stable features were related to geometric shape (22/24, 92%) and texture (37/99, 37%). A comprehensive list of all features is presented as supplementary information. After wavelet decomposition, 144 additional radiomics features were calculated for all 8 wavelet-filters, resulting in 27% (312/1152) of filtered features being stable. In 17 colorectal liver metastases from 17 patients, 25% of all extracted radiomics features (330/1322) demonstrated CCC-values greater than 0.85 in 1.5 T ADC-maps. Of these stable features, 36% (61/170) of unfiltered and 23% (269/1152) of wavelet-filtered features were stable in colorectal liver metastases. For 22 lung cancer lesions from 19 patients, 25% of all features (330/1322) showed stability matching our specified threshold. In contrast to colorectal liver metastases, only 16% (27/170) of unfiltered radiomics signatures were stable, while the percentage of stable wavelet-filtered features were 26% (303/1152).

**Table 1:** Stable features in ADC maps acquired at 1.5 T over different tumour-entities (i.e., 20 ovarian cancer lesions, 17 colorectal liver metastases, 22 lung cancer lesions).

Tumour type (1.5 T)	ADC Mean ± SD (10^−6^ mm^2^/s)	Stable features (CCC > 0.85)				
		Unfiltered			Wavelet filtered	ALL (unfiltered + wavelet)
		*Intensity*	*Shape*	*Texture*		
Ovarian	1086.2 ± 191.9	7/47	22/24	37/99	312/1152	378/1322 **(29%)**
Colorectal Liver	979.2 ± 420.9	8/47	20/24	33/99	269/1152	330/1322 **(25%)**
Lung	1340.2 ± 412.5	1/47	13/24	13/99	303/1152	330/1322 **(25%)**

**Figure 3:**
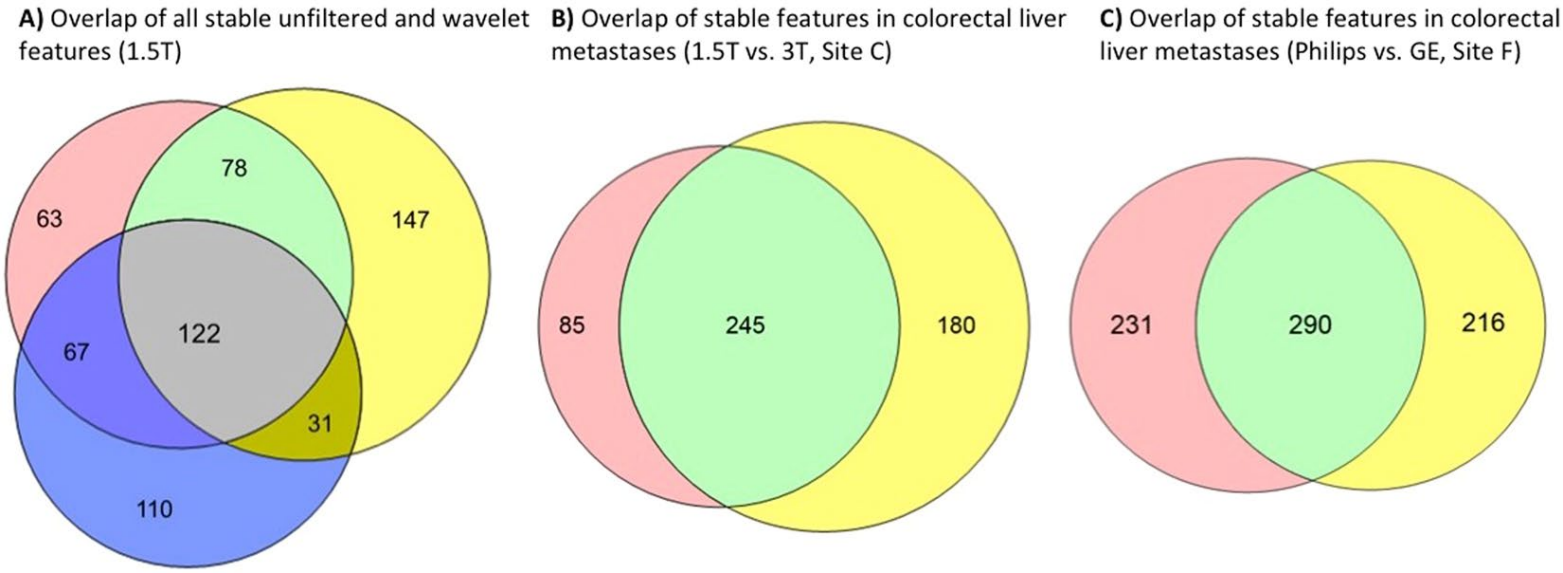
Overlapping results in feature stablility extracted from 1.5 T-MR images of (**A**) all tumour-entities (i.e., colorectal liver metastases (red), ovarian cancer (yellow), and lung cancer (blue)); derived from MR images of colorectal liver metastases (**B**) acquired at 1.5 T (red) and 3 T (yellow); and obtained from 3 T-MR images of colorectal liver metastases (**C**) acquired on a Philips Ingenia (red) and GE Discovery (yellow).

122 features (23 unfiltered and 99 wavelet-filtered) were regarded as stable in all three tumour entities and 298 features (49 unfiltered and 249 wavelet-filtered) were stable for at least 2 tumour-types on 1.5 T ADC maps (Fig. 3A, Supplementary Fig. S1). Statistically significant differences in CCC from all features (unfiltered and wavelet-filtered) were found between ovarian tumours and colorectal liver metastases (P < 0.0001), and between colorectal liver metastases and lung cancer (P = 0.0051) but not between ovarian and lung cancer (P = 0.56).

#### Magnetic field strength differences

The effects of magnetic field strength differences were analysed in ADC-maps acquired at 1.5 T and at 3 T in 17 and 13 patients with colorectal liver metastases, respectively. On ADC maps acquired at 3 T, 32% (425/1322) of radiomics features were stable over 13 segmented lesions from test-retest scans (Table 2). These consisted of 71/170 unfiltered features (i.e., 13/24 (54%) geometric shape features, 44/99 (44%) texture features, and 14/47 (30%) intensity features) and 355/1152 (29%) of wavelet-filtered features.

**Table 2:** Test-retest feature stability of colorectal liver metastases measured on 1.5 T (n = 17) and 3 T ADC maps (n = 13).

Magnetic field (Colorectal Liver)	ADC Mean ± SD (10^−6^ mm^2^/s)	ADC Median (10^−6^ mm^2^/s)	Stable features (CCC > 0.85)		
			Unfiltered	Wavelet filtered	ALL (unfiltered + wavelet)
1.5 T	979.2 ± 420.9	953.4	61/170	269/1152	330/1322 **(25%)**
3 T	1353.3 ± 409.8	1202.6	71/170	355/1152	425/1322 **(32%)**

No statistically significant differences were found in stability between features extracted from ADC maps acquired at 1.5 T and 3 T (P = 0.51). Correspondingly, 245 extracted features (42 unfiltered and 204 filtered) were shown to be stable in both populations, regardless of magnetic field strength (Fig. 3B, Supplementary Fig. S2). Furthermore, comparable mean and median ADC values were derived from both 1.5 T- and 3 T-ADC maps of the entire cohort (Table 2).

#### Cross-vendor differences

Vendor-specific subgroups were analysed within the dataset of colorectal liver metastases acquired at 3 T (site F). As shown in Table 3, feature stability of ADC maps acquired on a Philips Ingenia and a GE Discovery MR-system presented CCC-values > 0.85 for 521/1322 (39%) and 506/1322 (38%) features, respectively. No statistically significant differences were found in the number of features that exceeded the CCC-threshold (P = 0.49). However, 290 features (79 unfiltered and 211 filtered) presented high stability across both vendors’ test-retest data (Fig. 3C, Supplementary Fig. S3). Furthermore, 154 features (34 unfiltered, 120 filtered) were stable in both cross-vendor and in both 1.5 T and 3 T datasets.

**Table 3:** Test-retest feature stability of colorectal liver metastases measured on 3 T ADC maps acquired on a Philips Ingenia (n = 10) and GE Discovery (n = 8) MR systems at the same clinical centre.

Site F (Colorectal Liver, 3 T)	ADC Mean ± SD (10^−6^ mm^2^/s)	ADC Median (10^−6^ mm^2^/s)	Stable features (CCC > 0.85)		
			Unfiltered	Wavelet filtered	ALL (unfiltered + wavelet)
Philips	1237.9 ± 324.4	1129.8	106/170	415/1152	521/1322 **(39%)**
GE	1752.3 ± 395.6	1882.9	100/170	406/1152	506/1322 **(38%)**

A detailed description of stable features is described in Supplementary Tables S1-S4 for each subgroup and listed as supplementary information (online-only).

### Correlations between features and tumour volume

Across all tissue types and centres, the mean absolute of Spearman’s ***r*** (|***r***|) was 0.34 ± 0.23. The distribution of Spearman’s **r** values for all features can be seen in Supplementary Fig. 4. A total of 73 features were found to correlate highly with tumour volume (|***r***| > 0.8), 10 of which were shape features such as maximum diameter and surface, and the remaining 63 were made up of 7 texture features and their 56 wavelet filtered equivalents. A further 137 features correlate strongly (|***r***| > 0.6) with volume, of which 11 are texture features, two intensity histogram features, and one is a statistical feature, their associated 112 wavelet features, an additional shape feature, and 10 further wavelets of texture features. For all unfiltered features, Supplementary Fig. 5 shows the correlation between |***r***| and CCC for all tumour sites while Supplementary Fig. 6 details the strength of the correlations with tumour volume.

## Discussion

Within a multicentre trial, we present a method of data analysis to evaluate the stability of radiomics features derived from parametric MRI. This approach has shown that a substantial fraction of ADC-based radiomics features (25–29%) presented test-retest stability over a variety of tissues, MR-systems, and vendors. In addition, 122 features were stable over all tissues and could be regarded to be independent of tumour origin. These results regarding radiomics feature stability are in line with studies that analysed repeatability of absolute ADC values, a correlation that could be attributed to the low coefficient of variance (CoV) presented in these studies^5,18,19^. The methodology for stable feature selection and volume correlation presented in this analysis, together with the list of repeatable ADC radiomics features, facilitate the development of reliable MR-based radiomics signatures, and future clinical implementation across multiple centres. We therefore postulate that this method of analysis can be generalized to a larger field of quantifiable MR imaging features.

Shape features describe the volume contained within the segmentation, rendering it volume-dependent. Mismatch in shape features between test and retest scans could thus be attributed to differences in tumour segmentation and inter-observer variability although the same observer outlined the test-retest data in this study^20^. Especially in lung cancer, accurate tumour segmentation is complicated by respiratory motion and motion-related MR artefacts which vary between test-retest studies. Since DWI protocols in the lung were acquired during free-breathing, low stability of shape features is expected. However, these results were also present in radiomics analyses of CT-images where the acquisition is done in breath-hold^17^. Tumorous lesions in the liver and ovaries are less subjected to respiratory motion and as expected produced shape features with higher stability.

Texture features describe the pattern distribution of the voxels and quantify intra-tumour heterogeneity in all three dimensions^21^. As it takes into consideration the spatial relation to nearby voxels, stability across test-retest were similar to those of the shape features. In lung cancer, MR-related susceptibility artefacts are more common in DWI with distortions at the boundaries of tumour and air-filled lung tissue^11^. Hard transitions between tumours and normal lung tissue further complicate tumour segmentation as small delineation differences could have large impact on ADC measurements^20^. Nevertheless, radiomics features derived from lung cancer ADC maps achieved comparable reproducibility after wavelet-filtering, which can alleviate boundary inhomogeneity, justifying the use of ADC-based radiomics in multi-centre trials in lung cancer.

Intensity features in lung cancer also showed more variance compared to colorectal liver metastases and ovarian cancer. As the DWI protocol in lung cancer was acquired in free-breathing, variation in partial volume effects during test-retest are very likely to have been responsible for the high variation of the intensity features. There are several other reasons for variability in intensity feature stability in MRI. Unlike CT, variations in signal-intensity result from differences in RF coil sensitivity and coil placement during patient repositioning. Despite attempting to mitigate this by using ADC rather than absolute values of signal-intensity on high b-value images, intensity-features generally showed low stability. Although no treatment was administered to our patients between test-retest scans, eliminating the possibility of treatment-related physiological effects^3,22^, inflammatory processes associated with the tumour (e.g., lung-cancer atelectasis) and small molecular fluctuations of thermal diffusion, may also have affected the stability of the ADC intensity features^23^.

Stability of radiomics features was unaffected by differences in magnetic field strength, matching the field-independent nature of ADC^3^. In the literature, no significant difference has been reported between 1.5 T and 3 T ADC values measured in multiple organs^24,25^. However, higher mean CoVs have been reported in 3 T abdominal ADC^24^. Potentially, this variability is associated with increased difficulty in maintaining homogenous excitation pulses and gradient linearity at higher magnetic fields, and to the presence of artefacts related to magnetic susceptibility and eddy currents^25^.

Surprisingly, no statistically significant differences in feature stability were found between vendors, despite potential technical differences (e.g., imaging algorithms, shimming, fat suppression, and ADC reconstruction) (P = 0.49)^23,26^. Previously, studies have shown low longitudinal inter-vendor ADC variability when evaluating an ice/water phantom (CoV = 1–4%) and cancer patients (CoV ≤ 10%)^16,27,28^. High repeatability and low inter-scanner variation of ADC measurements could have a direct positive effect on radiomics feature stability.

Radiomics features derived from CT-images have been shown to have a prognostic value^9^. For CT, these included tumour intensity (‘energy’), texture (‘Grey-level non-uniformity’), wavelet (Grey-level non-uniformity HLH’) and shape (‘compactness’). These features were also highly stable in all tumour types in our data with CCC-values ranging from 0.89–0.99 (Fig. 1). This opens the possibility that similar radiomics features derived from ADC data might be useful in model-building, or in complementing the currently used method of detecting treatment-related changes by analysing absolute ADC metrics (i.e., histogram analysis of centiles, kurtosis and skewness)^18^.

Radiomics studies in CT and positron emission tomography have suggested that some reproducible features could be a surrogate of tumour volume^29,30^. This appears to hold true for some but certainly not the majority of the stable features presented in this research, and knowledge of which features correlate highly with volume is important for any radiomics study moving forward. Since radiomics should be purely quantitative imaging, no prognostic or diagnostic features should be excluded a priori, including simple ones such as volume or those that correlate with it.

Our analysis methods and underpinning study set-up had some limitations. Although efforts were made to minimise sources of variability by using a quality-assured standardized protocol and excluding b-values below 100 mm/s^2^ from ADC reconstruction to reduce perfusion-related MR-signal, the use of a standardised protocol within a multi-site study does not permit optimization of data from individual MR-systems^26^. Furthermore, DWI protocols did not include respiratory triggering or motion correction. Motion artefacts, predominantly seen in lung cancer patients and patients with colorectal liver metastases (site C, 3 T), had an adverse effect on ADC feature stability. However, for the colorectal liver metastases data acquired at 3 T, the protocol was adjusted from the one specified at 1.5 T to avoid specific image-artefacts^24^. For example, a larger bandwidth of 1500–2650 Hz/pixel was set to minimise geometric image distortion. In addition to standardised DWI acquisition, we did not specifically reduce ADC variability through post-processing to further improve the stability of radiomics features^31^. In a recent multi-centre study by Pathak *et al*., the percentage change in test-retest ADC measurements decreased from 21.1% to 2.7% in colorectal liver metastases using a standardization-strategy to account for measurement uncertainty (i.e., error modelling). This type of approach has the potential to further improve the stability of radiomics features. In addition, radiomics stability could benefit from improvements in tumour segmentation (i.e., reducing inter- and intra-observer variability) and image quality (i.e., increased signal-to-noise ratio and reduced image artefacts)^5,6,20,31^. Although we controlled for observer segmentation (same observer segmented each test-retest), image reconstruction and processing algorithms varied between centres and scanner vendors. Neither the assessment of differences between medical centres nor the number of reproducible features as a function of CCC cut-off (as performed in^17^) were included in this study owing to the small number of patients from each site and should be addressed in future work. However, good agreement in ADC-measurements between centres previously has been reported^27^. Also, image pre-processing can be regarded as another variable in the feature extraction workflow, and as such is also able to influence feature variability, so minimal pre-processing was performed, using common values from radiomics literature. Performing test-retest experiments are crucial in order to ensure that only stable features are selected for meaningful analysis and inclusion of parametric MR-radiomics as a clinical tool^15^.

In conclusion, we have presented the assessment of stability of radiomics features from parametric ADC based on standardized test-retest measurements. This methodology enables selection of stable features that quantitatively represent phenotypic features and enables the exciting use of high-quality radiomics analysis to attain reliable biomarkers complementary to other clinical/imaging data. As extracted ADC-based radiomics features are stable across multiple centres, tumour types, 1.5 T-3 T systems, and MR-vendors, this analysis can be widely included in multicentre trials. The implementation of such quantitative analysis of tumour phenotype will facilitate the development of diagnostic and theragnostic models that could help detect cancers earlier than the current standard, predict early treatment response and improve treatment decision-making towards personalized healthcare.

## Methods

### Patient population

As part of prospective clinical trials to qualify ADC imaging biomarkers and stability performed by the Quantitative Imaging in Cancer: Connecting Cellular Processes with Therapy (QuICConCePT) consortium (lung and liver) and the Cancer Research UK (ovary), sixty-one patients were included from 6 university hospitals across the UK, Italy, France, and the Netherlands (site A-C, and E-G). Included patients were diagnosed with either lung cancer stage III^18^, ovarian cancer (Winfield *et al*., personal communication), or colorectal liver metastases^32^, and had a minimum of two imaging sessions maximally 7 days apart before start of treatment. Patient cohorts are summarized in Table 4. This study was approved by the institutional medical ethics committee of each centre (Medical Ethical Committee VU University Medical Centre, Ethics Committee Humanitas Milan, INSERM Ethics Committee, University Research Ethics Committee at the University of Manchester, *Commissie Mensgebonden Onderzoek regio Arnhem – Nijmegen (WMO)* at the Radboud University Medical Centre Nijmegen, and the Research Ethics Committee for The Royal Marsden Hospital Sutton). Formal written informed consent was recorded for each participant and all data analyses were compliant to the Medical Research Involving Human Subjects Act (WMO).

**Table 4:** Main patient cohort characteristics.

Tumour site	Tumour type	Number	Age range	Treatment received
Lung	NSCLC/metastases	19	41–86	5 naïve, 14 previously treated
Liver	Colorectal metastases	30	44–77	No treatment within 6 months
Ovary	High grade serous	12	31–77	Naïve

### Image acquisition

Patients were scanned twice within 7 days before the start of treatment. In total, DWIs were acquired on 4 different MRI systems of 1.5 Tesla (T) and 3 different MRI systems of 3 T using a common scan protocol per tumour region (Table 5): on 1.5 T - GE Signa HDxt (site A), Philips Achieva DS (site B,G) Siemens Magnetom Avanto (site C,E), and on 3 T Siemens Magnetom Trio Tim (site C), Philips Ingenia (site F), and GE Discovery 750 w (site F). The applied MR-protocol was comprised of T1/T2-weighted images for anatomical imaging and diffusion-weighted sequences with three b-values. For DWI of each tumour-type, a common and quality-assured protocol was applied by all centres^27^ (Table 5). ADC maps were constructed by mono-exponential linear fitting of diffusion data. Images with b-values smaller than 100 s/mm^2^ were excluded to minimize components of blood perfusion in parametric ADC maps.

**Table 5:** Diffusion-weighted MR scan protocol. (*) Philips Ingenia and GE Discovery.

	Lung Cancer (site A, B, E, G)	Colorectal liver metastases (1.5 T) (site A, B, C, E)	Colorectal liver metastases (3 T) (site C, F*)	Ovarian cancer (site E)
Sequence	ss-EPI	ss-EPI	ss-EPI	ss-EPI
TR (ms)	≥8000	≥8000	5000	≥8000
TE (ms)	minimum	minimum	minimum	minimum
NSA	4	4	2–4	4
FOV (mm^2^)	380 × 273	380 × 380	380 × 273	332 × 380
Matrix	128 × 112	128 × 128	128 × 128	128 × 112
Bandwidth (Hz/px)	1400–1800	1400–1800	1500–2650	1400–1800
Slice thickness (mm)	5	5	5–6	6
Slice gap (mm)	0	0	0	0
Pixel size (mm^2^)	3 × 3	1.5 × 1.5	1.5 × 1.5	1.5 × 1.5
b-values	100, 500, 800	100, 500, 900	150, 400, 800	100, 500, 900
Fat saturation	yes	yes	yes	yes
Parallel imaging	yes	yes	yes	yes

### Segmentation

Volumes-of-interest (VOI) were manually delineated over all primary tumours and metastases on DWI images with high b-value. The gross tumour volume (GTV) was determined at central review by experienced radiologists (with a minimum of 2 years experience) using all diagnostic information available, and saved as binary masks. For each patient, the same observer segmented the same lesion in both test-retest images, while at the same time making sure that no large anatomical variations occurred. After voxel-wise rigid registration, tumour delineations on DWI were transferred to corresponding ADC maps. If ≥2 tumours were present, the 2 largest lesions were delineated while excluding cystic or necrotic regions from segmentations. The same lesion was delineated separately on retest-data while blinded from test-data. In total, 72 lesions were included for analysis. All segmentations were performed using OsiriX (Pixmeo SARL, Bernex, SUI), Mirada RX (Mirada Medical, Oxford, UK), or Adept (in-house software, Institute of Cancer Research, London, UK).

### Feature extraction

Radiomics features were retrospectively extracted from each VOI in the test-retest ADC dataset. ADC maps were pre-processed in two steps: (1) in order to reduce image noise and grey-level matrix (GLM) size, images were rescaled using a bin-size of 25 grey levels; (2) in order to standardise voxel size across all datasets, images were rescaled using a linearly resampled into isotropic voxel-sizes of 3 × 3 × 3 mm^3,33^. A total of 1322 radiomics features were obtained using an in-house developed software-toolbox in MATLAB 2014a (Mathworks, Natick, USA)^21^. These features included signal intensity features (n = 47), geometric features (n = 24), and texture features (n = 99), which respectively described the histogram-distribution of voxel intensity-values (i.e, first-order grey-level statistics, local intensity (Locint), and intensity histogram (IH) features), the 3D shape of delineated volumes, and the spatial distribution of fractal dimensions and voxel intensities using 6 texture matrices (i.e., grey-level co-occurrence (GLCM, 26 features)^34^, grey-level distance-zone (GLDZM, 16 features)^35^, grey-level run-length (GLRLM, 16 features)^5,36^, grey-level size-zone (GLSZM, 16 features)^37^, neighbouring grey-level dependence (NGLDM, 17 features)^38^, and neighbourhood grey-tone difference matrix (NGTDM, 5 features)^39^. Furthermore, 3D wavelet decompositions of the original image resulted in additional 1152 features focusing on different spatial frequency ranges within tumour values^9^.

A mathematical description of all features was previously published in^9,21,40^ and were presented as supplemental material with permission of the corresponding authors. Most features used in this study are in compliance with feature definitions as described by the Imaging Biomarker Standardization Initiative (IBSI). Where features differ, a note has been added specifying the difference.

### Statistical analysis

To select stable radiomics features, the pairwise concordance correlation coefficient (CCC) was calculated between data derived from the test and retest ADC images^41^. CCC-values range from −1 to +1 and describe the negative or positive agreement between 2 datasets. Based on previous work, features with a minimum CCC of 0.85 were regarded as statistically stable and robust^17,40,42^. Stability is defined as the closeness of agreement between measured quantity values obtained by replicate measurements performed under the same conditions (e.g., patient, scanner, imaging protocol)^18^. Statistical differences in stable features between tumour types, and between MR-systems with different magnetic field strengths were tested using Kruskal-Wallis 1-way ANOVA with Dunn’s correction for multiple testing. Differences between MR-systems from different vendors were tested for statistical significance using a Mann-Whitney test. All statistical analyses were performed using GraphPad Prism version 6.01 (GraphPad, USA). P-values < 0.05 were considered statistically significant.

### Feature correlation with tumour volume

Features with a constant value (or near-zero variance) across all images in the test dataset were excluded, and the remainder were examined for correlations with the tumour volume using Spearman’s rho statistic to estimate a rank-based measure of association. The Spearman coefficients of all unfiltered features were plotted against the feature stability as measured by the CCC for all tumour types and field strengths.

## Supplementary information

Supplementary material
