# Supplementary material for: Stability of radiomics features in apparent diffusion coefficient maps from a multi-centre test-retest trial
Source: Sci Rep. 2019 Mar 18;9:4800. doi: 10.1038/s41598-019-41344-5 (PMC6423042; doi:10.1038/s41598-019-41344-5)
Supplement: Supplementary file 1 — Supplementary material [file 41598_2019_41344_MOESM1_ESM.pdf]

Stability of radiomics features in apparent diffusion coefficient maps from a multi-centre test-retest trial: Supplementary Material

Jurgen Peerlings[1,2], Henry C. Woodruff[1,2], Jessica M. Winfield[3], Abdalla Ibrahim[1,2], Bernard E. Van Beers[4], Arend Heerschap[5], Alan Jackson[6], Joachim E. Wildberger[2], Felix M. Mottaghy[2,7], Nandita M. deSouza[3]†, Philippe Lambin[1,2]†

1. *The D-Lab, Department of Precision Medicine, GROW - School for Oncology and Developmental Biology, Maastricht University Medical Centre+, Maastricht, The Netherlands*
2. *Department of Radiology and Nuclear Medicine, GROW - School for Oncology and Developmental Biology, Maastricht University Medical Centre+, Maastricht, The Netherlands*
3. *Cancer Research UK Cancer Imaging Centre, The Institute of Cancer Research and Royal Marsden Hospital, Sutton, UK*
4. *Laboratory of Imaging Biomarkers, UMR 1149 Inserm - University Paris Diderot, Paris; Department of Radiology, Beaujon University Hospital Paris Nord, Clichy, France*
5. *Department of Radiology, Radboud University Nijmegen Medical Center, Nijmegen, NL*
6. *Wolfson Imaging Centre, Wolfson Molecular Imaging Centre, University of Manchester, 23 Palatine Rd, Withington, Greater Manchester, UK*
7. *Department of Nuclear Medicine, University Hospital RWTH Aachen University, Aachen, Germany*

(† both senior authors contributed equally)

Corresponding author: h.woodruff@maastrichtuniversity.nl

Supplementary Figure 1: Overlap in stable unfiltered, wavelet and all features between tumour entities at 1.5T (i.e., colorectal liver metastases (red), ovarian (yellow), and lung (blue) cancer)

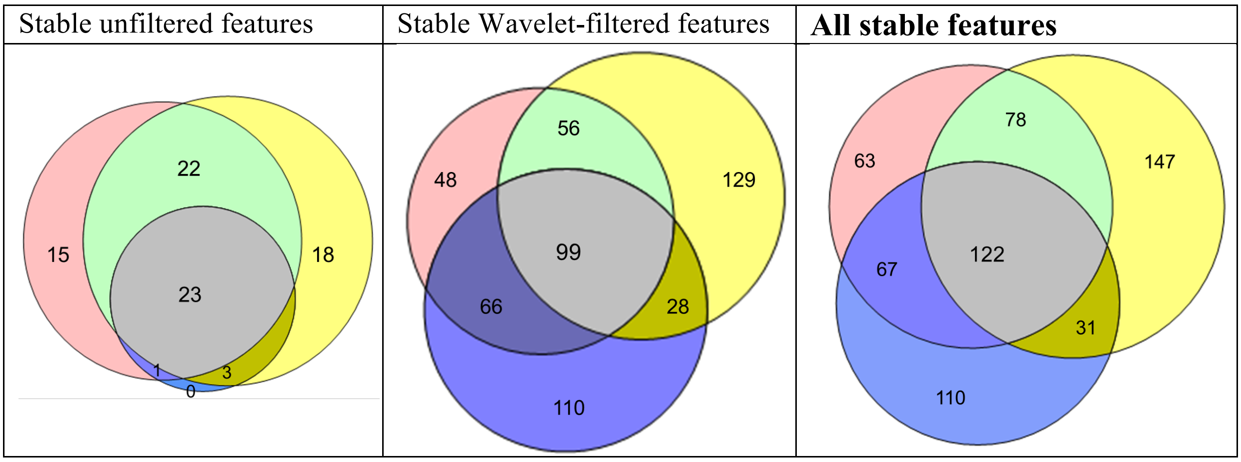

Supplementary Figure 2: Overlap in stable features between colorectal liver metastases at 1.5T (red) and 3T (yellow):

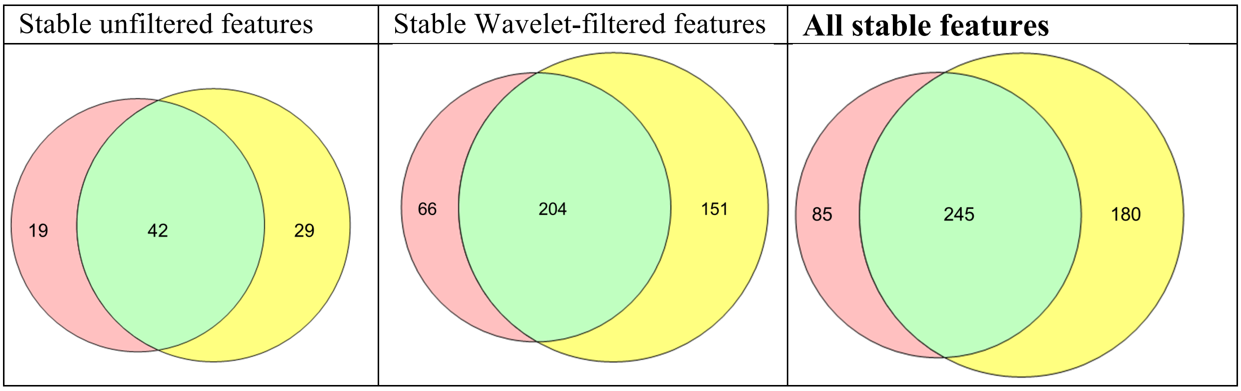

Supplementary Figure 3: Overlap in stable features between different MR-systems of Philips (red) and GE (yellow):

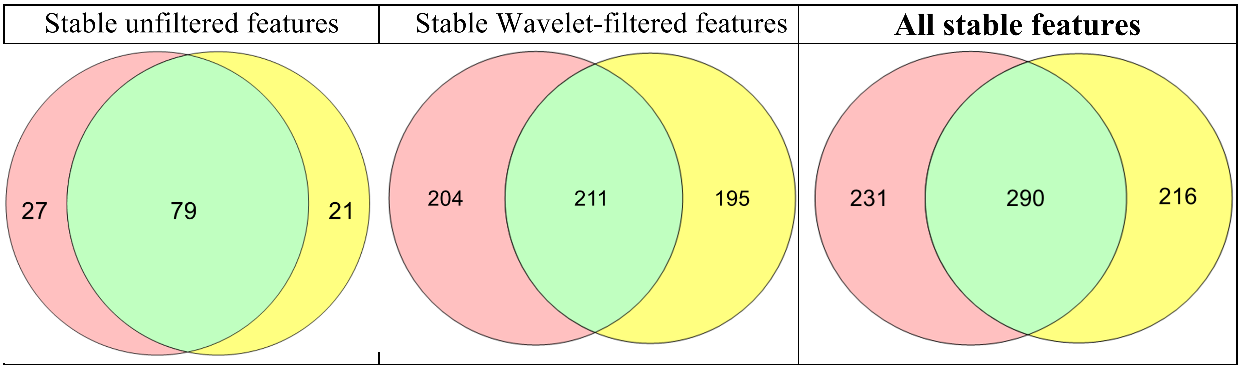

Supplementary Figure 4: Histogram of Spearman's r values correlating feature values with tumour volume:

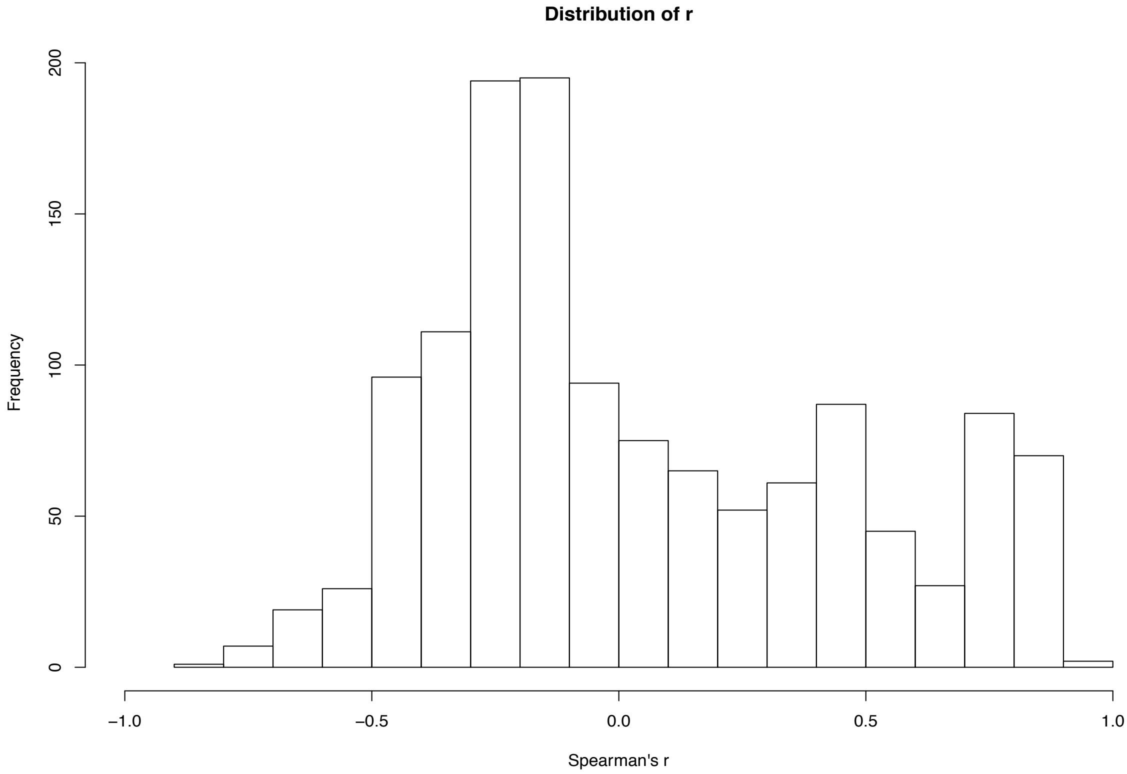

Supplementary Figure 5: Absolute value of Spearman's ***r*** plotted as a function of the CCC:

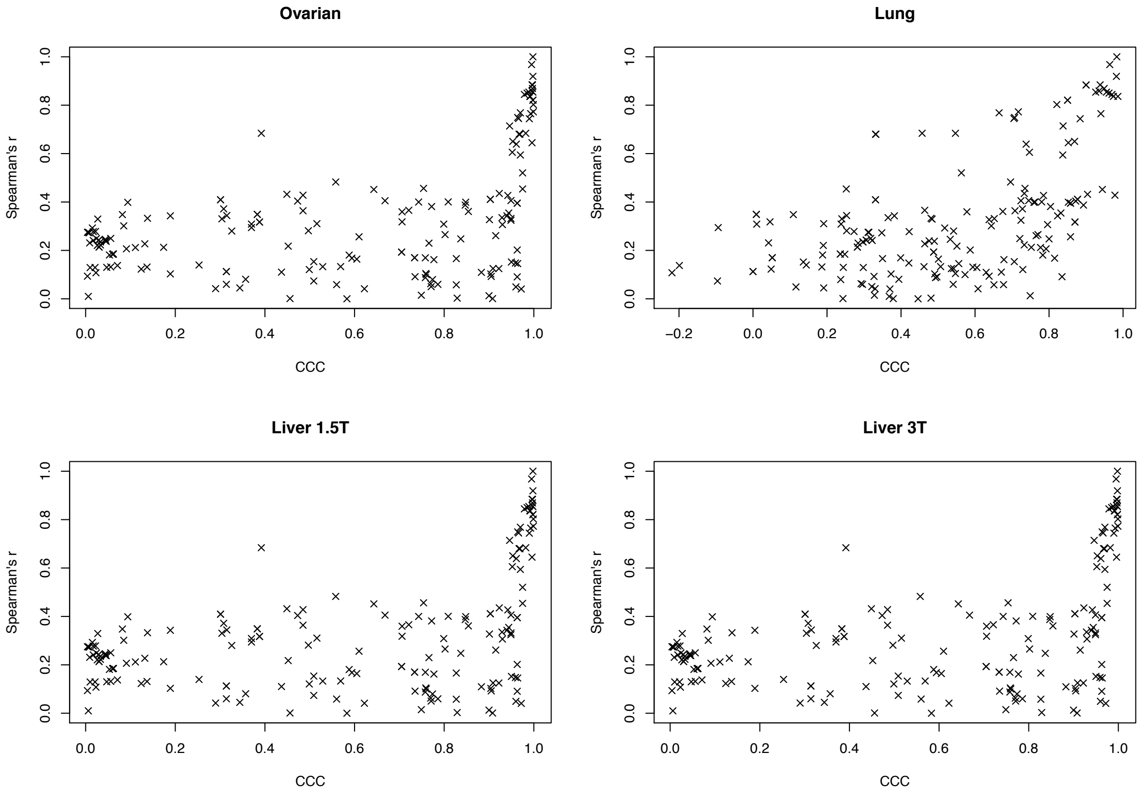

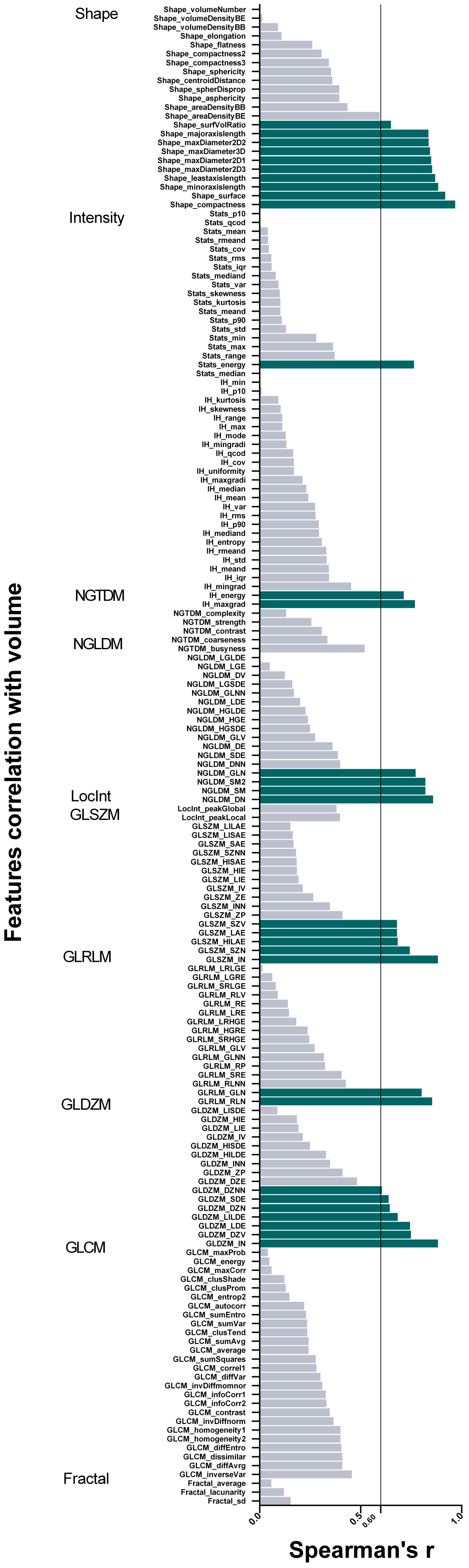

Supplementary Figure 6: Spearman correlationss between the radiomics features extracted from the test dataset for all patients. The threshold for strong correlation was set to ***r*** greater than 0.6.

Supplementary information 2: Mathematical description of extracted radiomics features.

Feature descriptions

* - Not described in IBSI feature definitions

Fractal Dimension features*

The Fractal Dimension (FD) of the image is computed as described by Al-Kadi and Watson (1). Given the FD processed image I, with N elements:

1. Average

$$average = \frac{1}{N}\sum I$$

2. Lacunarity

$$lacunarity = \frac{\frac{1}{N}\sum I^2}{\frac{1}{N^2}\sum I} - 1$$

3. Standard deviation

$$standard\ deviation = \left(\frac{1}{N-1}\sum (I-\bar{I})^2\right)^{1/2}$$

Where $\bar{I}$ is the mean of I.

First-order Grey-level statistics

First-order Grey-level statistics describe the distribution of grey values within the volume. Let X denote the three dimensional image matrix with N voxels, P the first order histogram, $P(i)$ the fraction of voxels with intensity level i and N_l the number of discrete intensity levels.

1. Energy

$$energy = \sum_{i=1}^{N} X(i)^2$$

Energy is also known as the sum of squares.

2. Entropy

$$entropy = \sum_{i=1}^{N_l} P(i)\log_2 P(i)$$

Note: Defined by IBSI as Intensity Histogram Entropy.

3. Kurtosis

$$kurtosis = \frac{\frac{1}{N}\sum_{i=1}^{N}(X(i) - \bar{X})^4}{\left(\frac{1}{N}\sum_{i=1}^{N}(X(i) - \bar{X})^2\right)^2}$$

where $\bar{X}$ is the mean of X.
Note: The IBSI feature definition implements excess kurtosis, where kurtosis is corrected by -3, yielding 0 for normal distributions. The kurtosis presented above is not corrected, yielding a value 3 higher than the IBSI kurtosis.

4. Maximum
The maximum grey value of X.

$$maximum = \max(X)$$

5. Mean
The mean grey value of X.

$$mean = \frac{1}{N}\sum_{i=1}^{N} X(i)$$

Mean ADC values of the entire cohort was calculated over the mean of each lesion

6. Mean absolute deviation
The mean of the absolute deviations of all voxel intensities around the mean intensity value.

$$mean\ absolute\ deviation = \frac{1}{N}\sum_{i=1}^{N}\left|X(i) - \bar{X}\right|$$

where $\bar{X}$ is the mean of X.

7. Median

The sample median of X, or the 50th percentile of X.
Median ADC values of the entire cohort was calculated over the mean of each lesion

8. Minimum
The minimum intensity value of X.

$$minimum = \min(X)$$

9. Range
The range of intensity values of X.

$$range = \max(X) - \min(X)$$

10. Root mean square (RMS)
The quadratic mean, or the square root of the mean of squares of all voxel intensities.

$$RMS = \sqrt{\frac{\sum_{i}^{N} X(i)^2}{N}}$$

11. Skewness

$$skewness = \frac{\frac{1}{N}\sum_{i=1}^{N}(X(i)-\bar{X})^3}{\left(\sqrt{\frac{1}{N}\sum_{i=1}^{N}(X(i)-\bar{X})^2}\right)^3}$$

where $\bar{X}$ is the mean of X.

12. Standard deviation *

$$standard\ deviation = \left(\frac{1}{N-1}\sum_{i=1}^{N}(X(i)-\bar{X})^2\right)^{1/2}$$

where $\bar{X}$ is the mean of X.

13. Robust mean absolute deviation

The mean absolute deviation (0) of only those voxels in X with a grey value between the 10th and 90th percentile.

14. 10th percentile
The 10th percentile of X, a robust alternative to the minimum grey value (8).

15. 90th percentile
The 90th percentile of X, a robust alternative to the maximum grey value (4).

16. Interquartile range
The interquartile range is defined as the 75th minus the 25th percentile of X.

17. Uniformity

$$uniformity = \sum_{i=1}^{N_l} P(i)^2$$

Note: Defined by IBSI as Intensity Histogram Uniformity.

18. Variance

$$variance = \frac{1}{N-1}\sum_{i=1}^{N}(X(i)-\bar{X})^2$$

where $\bar{X}$ is the mean of X. Variance is the square of the standard deviation (12).

Intensity histogram features

Intensity histogram features describe the distribution of grey values within the volume, after discretization into intensity level bins was applied. Let:

$X_d = \{X_{d,1}, X_{d,2}, \dots, X_{d,N_v}\}$ be the set of discretized intensity values of the N_v voxels in the volume of interest,
$H = \{n_1, n_2, \dots\}$ be the histogram with frequency count n_i of each discretized intensity level i in X_d,

N_g be the number of discretized intensity values (bins) in the volume of interest,
p_i be the occurrence probability for each bin i of the histogram N_g, where $p_i = n_i/N_v$.

1. Coefficient of variance (cov)

$$cov = \frac{\text{standard deviation}}{\text{mean}}$$

2. Energy

$$energy = \sum_{j=1}^{N_v} X_d(j)^2$$

Energy is also known as the sum of squares.

3. Entropy

$$entropy = -\sum_{i=1}^{N_g} P(i)\log_2 P(i)$$

4. Interquartile range (iqr)

$$IQR = P_{75} - P_{25}$$

where P_{25} and P_{75} are the 25th and 75th percentile of X_d, respectively.

5. Kurtosis

$$kurtosis = \frac{\frac{1}{N_v}\sum_{j=1}^{N_v}(X_d(j) - \bar{X}_d)^4}{\left(\frac{1}{N_v}\sum_{j=1}^{N_v}(X_d(j) - \bar{X}_d)^2\right)^2}$$

where $\bar{X}_d$ is the mean of X_d.

6. Maximum

The maximum discretized intensity value of X_d.

$$maximum = \max(X_d)$$

7. Maximum histogram gradient (maxgrad)

$$maxgrad = \max(H')$$

Where H' is the histogram gradient, defined as:

$$H' = \{H(2) - H(1), \ldots, \frac{H(i+1) - H(i-1)}{2}, \ldots, H(N_g) - H(N_g - 1)\}$$

8. Maximum histogram gradient intensity level (maxgradi)

The discretized intensity level i corresponding to the maximum histogram gradient.

9. Mean

The mean discretized intensity value of X_d.

$$mean = \frac{1}{N_v}\sum_{j=1}^{N_v} X_d(j)$$

10. Mean absolute deviation (meand)

The mean of the absolute deviations of all discretized intensity levels around the mean of X_d.

$$meand = \frac{1}{N_v}\sum_{j=1}^{N_v}|X_d(j) - \overline{X_d}|$$

where $\bar{X}_d$ is the mean of X_d.

11. Median

The sample median of X_d or the 50th percentile of X_d.

12. Median absolute deviation (mediand)

The dispersion from the median of X_d.

$$mediand = \frac{1}{N_v}\sum_{j=1}^{N_v}|X_d(j) - M|$$

where M is the median of X_d.

13. Minimum

The minimum discretized intensity value of X_d.

$$minimum = \min(X_d)$$

14. Minimum histogram gradient (mingrad)

$$mingrad = \min(H')$$

Where H' is the histogram gradient, defined as:

$$H' = \{H(2) - H(1), \dots, \frac{H(i+1) - H(i-1)}{2}, \dots, H(N_g) - H(N_g - 1)\}$$

15. Minimum histogram gradient intensity level (mingradi)

The discretized intensity level i corresponding to the minimum histogram gradient.

16. Mode

The mode of X_d is the most frequently occurring discretized image level present. In case multiple bins have the highest count n_i, the mode is the smallest of those values.

17. Uniformity

$$uniformity = \sum_{i=1}^{N_g} P(i)^2$$

Note: Defined by IBSI as Intensity Histogram Uniformity.

18. Range

The range of bins in the histogram, i.e. the width of the histogram.

$$range = \max(X_d) - \min(X_d)$$

19. Root mean square (RMS):

$$RMS = \sqrt{\frac{\sum_{j=1}^{N_v} X_d(j)^2}{N_v}}$$

20. Robust mean absolute deviation (rmeand)

Similar to mean absolute deviation, but in this case only considering the set of intensity levels in the range between the 10th and 90th percentile of X_d.

$$rmeand = \frac{1}{N_{10-90}} \sum_{j=1}^{N_{10-90}} \left| X_{d,10-90}(j) - \bar{X}_{d,10-90} \right|$$

where X_{10-90} represents the set of N_{10-90} voxels in X_d whose discretized intensity levels fall within the range of the 10th till the 90th percentile of X_d.

21. Skewness

$$skewness = \frac{\frac{1}{N_v}\sum_{j=1}^{N_v}(X_d(j) - \bar{X}_d)^3}{\left(\sqrt{\frac{1}{N_v}\sum_{j=1}^{N_v}(X_d(j) - \bar{X}_d)^2}\right)^3}$$

22. Standard deviation

$$standard\ deviation = \left(\frac{1}{N_v - 1}\sum_{j=1}^{N_v}(X_d(j) - \bar{X}_d)^2\right)^{1/2}$$

23. Variance

The variance of X_d.

$$variance = \frac{1}{N_v - 1}\sum_{j=1}^{N_v}(X_d(j) - \overline{X_d})^2$$

where $\overline{X_d}$ is the mean of X_d.

24. 10th percentile

The 10th percentile of X_d.

25. 90th percentile

The 90th percentile of X_d.

26. Quartile coefficient of dispersion (qcod)

The quartile coefficient of dispersion is a robust alternative to the coefficient of variance.

$$qcod = \frac{P_{75} - P_{25}}{P_{75} + P_{25}}$$

where P_{25} and P_{75} are the 25th and 75th percentile of X_d, respectively.

*Local Intensity features **

Local Intensity (LocInt) features are defined based on local intensity values around a center voxel (2).

1. Local intensity peak

Mean intensity level in a 1 cm^3 spherical volume, centered on the voxel with the maximum intensity level in the volume of interest. In case multiple voxels contain the maximum intensity level, the highest mean intensity level of all spherical volumes is used.

2. Global Intensity peak

Similar to local intensity peak, but in this case the mean intensity level in a 1 cm^3 spherical volume is calculated for every voxel in the volume of interest. The highest mean intensity level of all spherical volumes is selected as the global intensity peak feature.

Geometric features

Geometric features describe the shape and size of the volume of interest. Let V be the volume and A the surface area of the volume of interest. Let N be the total number of voxels, $X = \{\vec{X}_1, \vec{X}_2, \dots, \vec{X}_N\}$ the set of N Cartesian coordinate vectors and $I = \{I_1, I_2, \dots, I_N\}$ the corresponding intensity values.

1. Asphericity

$$asphericity = \left(\frac{1}{36\pi}\frac{A^3}{V^2}\right)^{\frac{1}{3}} - 1$$

2. Centroid distance

The centroid distance is the Euclidean distance between the geometric centroid (C_g) and the centroid weighing each voxel by its intensity value (C_i). The centroid distance is a measure of how close the high intensity values are to the geometric center.

$$C_g = \frac{1}{N}\sum_{i=1}^{N} \vec{X}_i$$

$$C_i = \frac{\sum_{i=1}^{N} I\vec{X}_i}{\sum_{i=1}^{N} I}$$

$$centroid\ distance = \left\|C_g - C_i\right\|$$

3. Compactness 1

Compactness is a measure of how much the volume resembles a sphere, as described by Aerts et al. (3).

$$compactness\ 1 = \frac{V}{\sqrt{\pi}A^{\frac{2}{3}}}$$

4. Compactness 2

$$compactness\ 2 = 36\pi\frac{V^2}{A^3}$$

5. Compactness 3

$$compactness\ 3 = \frac{V}{\sqrt{\pi}A^{\frac{3}{2}}}$$

A dimensionless alternative to Compactness (3), as described by Aerts et al. (3).

6. Maximum diameter

The maximum diameter is the largest pairwise difference between voxels on the surface of the volume, in 3D and for each plane separately. The following diameters are calculated:

a. The maximum three-dimensional tumor diameter.

b. The maximum two-dimensional diameter of all transversal planes.

c. The maximum two-dimensional diameter of all sagittal planes.
d. The maximum two-dimensional diameter of all coronal planes.

7. Major axis length

Axis lengths are measures of the extent of the volume along its three principle axis. Principle component analysis (PCA) on the x, y and z coordinates of all voxels within the volume is used to determine the three orthogonal eigenvectors and corresponding eigenvalues (λ_{max}, λ_{minor}, λ_{min}). The major axis length is the largest eigenvalue (λ_{max}) as determined by PCA.

8. Minor axis length

The largest eigenvalue (λ_{minor}) as determined by PCA.

9. Least axis length

The smallest eigenvalue (λ_{min}) as determined by PCA.

10. Elongation

$$elongation = \frac{\lambda_{minor}}{\lambda_{max}}$$

11. Flatness

$$flatness = \frac{\lambda_{min}}{\lambda_{max}}$$

12. Spherical disproportion (4)

Spherical disproportion is a measure of how much the volume resembles a sphere.

$$spherical\ disproportion = \frac{A}{4\pi R^2}$$

Where A is the surface area and R is the radius of a sphere with the same volume as the tumor, obtained through:

$$R = \sqrt[3]{\frac{3V}{4\pi}}$$

13. Sphericity (4)

Sphericity is a measure of how much the volume resembles a sphere.

$$sphericity = \frac{\pi^{\frac{1}{3}}(6V)^{\frac{2}{3}}}{A} = \frac{(36\pi V^2)^{\frac{1}{3}}}{A}$$

14. Surface area

The surface area is calculated by triangulation (i.e. dividing the surface into connected triangles, which define the isosurface enclosing the volume) and is defined as:

$$surface\ area = \sum_{i=1}^{N} \frac{1}{2} |a_i b_i \times a_i c_i|$$

Where N is the total number of triangles covering the surface and a, b and c are edge vectors of the triangles.

15. Surface to volume ratio

$$surface\ to\ volume\ ratio = \frac{A}{V}$$

16. Volume

The volume is defined as the number of voxels within the volume multiplied by the voxel volume.

$$volume = Nv$$

Where v is the volume of a single voxel.

Note: In the IBSI feature definitions, a more precise approximation of the volume is used. That method uses tetrahedrons consisting of the origin and faces in the ROI. Although the method implemented here overestimates the volume, especially in small volumes, the difference will be negligible in large ROIs.

Grey-Level Co-Occurrence Matrix based features

Grey-level co-occurrence matrix (GLCM) based features, as originally described by Haralick et al (5). A normalized GLCM is defined as $P(i,j;\delta,\alpha)$, a matrix with size $N_g \times N_g$ describing the second-order joint probability function of an image, where the (i,j)th element represents the number of times the combination of intensity levels i and j occur in two pixels in the image, that are separated by a distance of δ pixels in direction α, and N_g is the maximum discrete intensity level in the image. Let:

$P(i,j)$ be the normalized (i.e. $\sum P(i,j) = 1$) co-occurrence matrix, generalized for any δ and α ,

$p_x(i) = \sum_{j=1}^{N_g} P(i,j)$,

$p_y(j) = \sum_{i=1}^{N_g} P(i,j)$,

μ_x be the mean of p_x, where $\mu_x = \sum_{i=1}^{N_g}\sum_{j=1}^{N_g} iP(i,j)$

μ_y be the mean of p_y, where $\mu_y = \sum_{i=1}^{N_g}\sum_{j=1}^{N_g} jP(i,j)$

σ_x be the standard deviation of p_x, where $\sigma_x^2 = \sum_{i=1}^{N_g}\sum_{j=1}^{N_g} P(i,j)(i-\mu_x)^2$

σ_y be the standard deviation of p_y, where $\sigma_y^2 = \sum_{i=1}^{N_g}\sum_{j=1}^{N_g} P(i,j)(j-\mu_y)^2$

$p_{x+y}(k) = \sum_{i=1}^{N_g}\sum_{j=1}^{N_g} P(i,j),\ i+j=k,\ k=2,3,\dots,2N_g,$

$p_{x-y}(k) = \sum_{i=1}^{N_g}\sum_{j=1}^{N_g} P(i,j),\ |i-j|=k,\ k=0,1,\dots,N_g-1,$

$HXY1 = -\sum_{i=1}^{N_g}\sum_{j=1}^{N_g} P(i,j)\ln(p_x(i)p_y(j))$,

$HXY2 = -\sum_{i=1}^{N_g}\sum_{j=1}^{N_g} p_x(i)p_y(j)\ln(p_x(i)p_y(j))$.

$HX = -\sum p_x \ln(p_x)$

$HY = -\sum p_y \ln(p_y)$

1. Average (μ)

$$average\ (\mu) = \frac{\sum_{i=1}^{N_g}\sum_{j=1}^{N_g}(i+j)P(i,j)}{2}$$

Note that for a symmetrical GLCM, $\mu = \mu_x = \mu_y$.

2. Autocorrelation

$$autocorrelation = \sum_{i=1}^{N_g} \sum_{j=1}^{N_g} ijP(i,j)$$

3. Cluster Prominence

$$cluster\ prominence = \sum_{i=1}^{N_g} \sum_{j=1}^{N_g} \left[i + j - \mu_x - \mu_y\right]^4 P(i,j)$$

4. Cluster Shade

$$cluster\ shade = \sum_{i=1}^{N_g} \sum_{j=1}^{N_g} \left[i + j - \mu_x - \mu_y\right]^3 P(i,j)$$

5. Cluster Tendency

$$cluster\ tendency = \sum_{i=1}^{N_g} \sum_{j=1}^{N_g} \left[i + j - \mu_x - \mu_y\right]^2 P(i,j)$$

6. Contrast (6)

$$contrast = \sum_{i=1}^{N_g} \sum_{j=1}^{N_g} |i - j|^2 P(i,j) = \sum_{k=0}^{N_g - 1} k^2\, p_{x-y}(k)$$

7. Correlation

$$correlation = \frac{\sum_{i=1}^{N_g} \sum_{j=1}^{N_g} ijP(i,j) - \mu_x \mu_y}{\sigma_x \sigma_y}$$

8. Difference Average (μ_{x-y})

$$difference\ average\ (\mu_{x-y}) = \sum_{k=0}^{N_g - 1} kp_{x-y}$$

9. Difference Entropy

$$difference\ entropy = -\sum_{i=0}^{N_g - 1} P_{x-y}(i) \log_2\left[P_{x-y}(i)\right]$$

10. Difference Variance

$$difference\ variance = \sum_{i=0}^{N_g - 1} (i - \mu_{x-y})^2 P_{x-y}(i)$$

11. Dissimilarity

$$dissimilarity = \sum_{i=1}^{N_g} \sum_{j=1}^{N_g} |i - j| P(i,j)$$

12. Energy (7)

$$energy = \sum_{i=1}^{N_g}\sum_{j=1}^{N_g}[P(i,j)]^2$$

This feature is also called Angular Second Moment (ASM) and Uniformity (6).

13. Entropy (H)

$$entropy\ (H) = -\sum_{i=1}^{N_g}\sum_{j=1}^{N_g}P(i,j)\log_2[P(i,j)]$$

14. Homogeneity 1

$$homogeneity\ 1 = \sum_{i=1}^{N_g}\sum_{j=1}^{N_g}\frac{P(i,j)}{1+|i-j|}$$

This feature is also called Inverse Difference (6).

15. Homogeneity 2 (7)

$$homogeneity\ 2 = \sum_{i=1}^{N_g}\sum_{j=1}^{N_g}\frac{P(i,j)}{1+|i-j|^2}$$

This feature is also called Inverse Difference Moment (6).

16. Informational measure of correlation 1 (IMC1)

$$IMC1 = \frac{H-HXY1}{\max\{HX,HY\}}$$

Where H is the entropy (13).

17. Informational measure of correlation 2 (IMC2)

$$IMC2 = \sqrt{1-e^{-2(HXY2-H)}}$$

Where H is the entropy (13).

18. Inverse Difference Moment Normalized (IDMN)

$$IDMN = \sum_{i=1}^{N_g}\sum_{j=1}^{N_g}\frac{P(i,j)}{1+\left(\frac{|i-j|^2}{N_g^2}\right)}$$

19. Inverse Difference Normalized (IDN)

$$IDN = \sum_{i=1}^{N_g}\sum_{j=1}^{N_g}\frac{P(i,j)}{1+\left(\frac{|i-j|}{N_g}\right)}$$

20. Inverse variance

$$inverse\ variance = \sum_{i=1}^{N_g}\sum_{j=1}^{N_g}\frac{P(i,j)}{|i-j|^2}\quad ,i\neq j$$

21. Maximal Correlation Coefficient

$$maximal\ correlation\ coefficient = \sqrt{\text{second largest eigenvalue of } Q}$$

$$Q = \sum_{k=1}^{N_g} \frac{P(i,k)P(j,k)}{p_x(i)p_y(k)}$$

22. Maximum Probability

$$maximum\ probability = \max\{P(i,j)\}$$

23. Sum average (SA)

$$sum\ average\ (SA) = \sum_{i=2}^{2N_g} \left[iP_{x+y}(i)\right]$$

24. Sum entropy

$$sum\ entropy = -\sum_{i=2}^{2N_g} P_{x+y}(i) \log_2\left[P_{x+y}(i)\right]$$

25. Sum variance

$$sum\ variance = \sum_{i=2}^{2N_g} (i - SA)^2 P_{x+y}(i)$$

26. Variance (sum of squares)

$$variance = \sum_{i=1}^{N_g}\sum_{j=1}^{N_g} (i - \mu)^2 P(i,j)$$

Grey-Level Run-Length matrix based features

Grey-level run-length matrix (GLRLM) based features, as described by Galloway et al. (8). Run length metrics quantify grey level runs in an image. A grey level run is defined as the length in number of pixels, of consecutive pixels that have the same grey level value. In a grey level run length matrix $p(i,j|\theta)$, the (i,j)th element describes the number of times j a grey level i appears consecutively in the direction specified by θ. Let:

$p(i,j)$ be the (i,j)th entry in the given run-length matrix p, generalized for any direction θ,

N_g the number of discrete intensity values in the image,

N_r the maximum run length,

N_s the total number of runs, where $N_s = \sum_{i=1}^{N_g}\sum_{j=1}^{N_r} p(i,j)$,

p_r the sum distribution of the number of runs with run length j, where $p_r(j) = \sum_{i=1}^{N_g} p(i,j)$,

p_g the sum distribution of the number of runs with grey level i, where $p_g(i) = \sum_{j=1}^{N_r} p(i,j)$,

N_p the number of voxels in the image, where $N_p = \sum_{j=1}^{N_r} jp_r$,

$p_n(i,j)$ the normalized run-length matrix, where $p_n(i,j) = \frac{p(i,j)}{N_s}$,

μ_r the mean run length, where $\mu_r = \sum_{i=1}^{N_g} \sum_{j=1}^{N_r} j p_n(i,j)$,

μ_g the mean grey level, where $\mu_g = \sum_{i=1}^{N_g} \sum_{j=1}^{N_r} i p_n(i,j)$.

1. Short Run Emphasis (SRE)

$$SRE = \frac{1}{N_s} \sum_{j=1}^{N_r} \frac{p_r}{j^2}$$

2. Long Run Emphasis (LRE)

$$LRE = \frac{1}{N_s} \sum_{j=1}^{N_r} j^2 p_r$$

3. Grey Level Non-Uniformity (GLN)

$$GLN = \frac{1}{N_s} \sum_{i=1}^{N_g} p_g^2$$

4. Grey Level Non-Uniformity Normalized (GLNN)

$$GLNN = \frac{1}{N_s^2} \sum_{i=1}^{N_g} p_g^2$$

5. Run Length Non-Uniformity (RLN)

$$RLN = \frac{1}{N_s} \sum_{j=1}^{N_r} p_r^2$$

6. Run Length Non-Uniformity Normalized (RLNN)

$$RLNN = \frac{1}{N_s^2} \sum_{j=1}^{N_r} p_r^2$$

7. Run Percentage (RP)

$$RP = \frac{N_s}{N_p}$$

8. Low Grey Level Run Emphasis (LGRE)

$$LGRE = \frac{1}{N_s} \sum_{i=1}^{N_g} \frac{p_g}{i^2}$$

9. High Grey Level Run Emphasis (HGRE)

$$HGRE = \frac{1}{N_s} \sum_{i=1}^{N_g} i^2 p_g$$

10. Short Run Low Grey Level Emphasis (SRLGE)

$$SRLGE = \frac{1}{N_s}\sum_{i=1}^{N_g}\sum_{j=1}^{N_r}\frac{p(i,j)}{i^2 j^2}$$

11. Short Run High Grey Level Emphasis (SRHGE)

$$SRHGE = \frac{1}{N_s}\sum_{i=1}^{N_g}\sum_{j=1}^{N_r}\frac{p(i,j)i^2}{j^2}$$

12. Long Run Low Grey Level Emphasis (LRLGE)

$$LRLGE = \frac{1}{N_s}\sum_{i=1}^{N_g}\sum_{j=1}^{N_r}\frac{p(i,j)j^2}{i^2}$$

13. Long Run High Grey Level Emphasis (LRHGE)

$$LRHGE = \frac{1}{N_s}\sum_{i=1}^{N_g}\sum_{j=1}^{N_r}p(i,j)i^2 j^2$$

14. Grey level variance (GLV)

$$GLV = \sum_{i=1}^{N_g}\sum_{j=1}^{N_r}(i-\mu_g)^2 p_n(i,j)$$

15. Run length variance (RLV)

$$RLV = \sum_{i=1}^{N_g}\sum_{j=1}^{N_r}(j-\mu_r)^2 p_n(i,j)$$

16. Run entropy (RE) (9)

$$RE = -\sum_{i=1}^{N_g}\sum_{j=1}^{N_r}p_n(i,j|\theta)\log_2[p_n(i,j)]$$

Grey-Level size-zone matrix based features

Grey-level size-zone matrix (GLSZM) based features, as described by Thibault et al. (10, 11). A grey level size-zone matrix describes the amount of homogeneous connected areas within the volume, of a certain size and intensity. The (i,j)th entry of the GLSZM $p(i,j)$ is the number of connected areas of grey level (i.e. intensity value) i and size j. GLSZM features therefore describe homogeneous areas within the tumor volume, describing tumor heterogeneity at a regional scale (12). Let:
$p(i,j)$ be the (i,j)th entry in the given GLSZM p,
N_g the number of discrete intensity values in the image,
N_z the size of the largest, homogeneous region in the volume of interest,
N_s the total number of homogeneous regions (zones), where $N_s = \sum_{i=1}^{N_g}\sum_{j=1}^{N_z}p(i,j)$,
p_z the sum distribution of the number of zones with size j, where $p_z(j) = \sum_{i=1}^{N_g}p(i,j)$,

p_g the sum distribution of the number of zones with grey level i, where $p_g(i) = \sum_{j=1}^{N_z} p(i,j)$,
N_p the number of voxels in the image, where $N_p = \sum_{j=1}^{N_z} jp_z$,
$p_n(i,j)$ the normalized size-zone matrix, where $p_n(i,j) = \frac{p(i,j)}{N_s}$,
μ_z the mean zone size, where $\mu_z = \sum_{i=1}^{N_g} \sum_{j=1}^{N_z} jp_n(i,j|\theta)$,
μ_g the mean grey level, where $\mu_g = \sum_{i=1}^{N_g} \sum_{j=1}^{N_z} ip_n(i,j|\theta)$.

1. Small area Emphasis (SAE)

$$SAE = \frac{1}{N_s} \sum_{j=1}^{N_z} \frac{p_z}{j^2}$$

2. Large area Emphasis (LAE)

$$LAE = \frac{1}{N_s} \sum_{j=1}^{N_z} j^2 p_z$$

3. Intensity Non-Uniformity (IN)

$$IN = \frac{1}{N_s} \sum_{i=1}^{N_g} p_g^2$$

4. Intensity Non-Uniformity Normalized (INN)

$$INN = \frac{1}{N_s^2} \sum_{i=1}^{N_g} p_g^2$$

5. Size-zone Non-Uniformity (SZN)

$$SZN = \frac{1}{N_s} \sum_{j=1}^{N_z} p_z^2$$

6. Size-zone Non-Uniformity Normalized (SZNN)

$$SZNN = \frac{1}{N_s^2} \sum_{j=1}^{N_z} p_z^2$$

7. Zone Percentage (ZP)

$$ZP = \frac{N_s}{N_p}$$

8. Low intensity Emphasis (LIE)

$$LIE = \frac{1}{N_s} \sum_{i=1}^{N_g} \frac{p_g}{i^2}$$

9. High intensity Emphasis (HIE)

$$HIE = \frac{1}{N_s}\sum_{i=1}^{N_g} i^2 p_g$$

10. Low intensity small area Emphasis (LISAE)

$$LISAE = \frac{1}{N_s}\sum_{i=1}^{N_g}\sum_{j=1}^{N_z}\frac{p(i,j)}{i^2 j^2}$$

11. High intensity small area Emphasis (HISAE)

$$HISAE = \frac{1}{N_s}\sum_{i=1}^{N_g}\sum_{j=1}^{N_z}\frac{p(i,j)i^2}{j^2}$$

12. Low intensity large area Emphasis (LILAE)

$$LILAE = \frac{1}{N_s}\sum_{i=1}^{N_g}\sum_{j=1}^{N_z}\frac{p(i,j)j^2}{i^2}$$

13. High intensity large area Emphasis (HILAE)

$$HILAE = \frac{1}{N_s}\sum_{i=1}^{N_g}\sum_{j=1}^{N_z} p(i,j)i^2 j^2$$

14. Intensity variance (IV)

$$IV = \sum_{i=1}^{N_g}\sum_{j=1}^{N_z}(i-\mu_g)^2 p_n(i,j)$$

15. Size-zone variance (SZV)

$$SZV = \sum_{i=1}^{N_g}\sum_{j=1}^{N_z}(j-\mu_z)^2 p_n(i,j)$$

16. Zone entropy (ZE)

$$ZE = \sum_{i=1}^{N_g}\sum_{j=1}^{N_z} p_n(i,j)\log_2[p_n(i,j)]$$

Grey-Level distance-zone matrix based features

Grey-level distance-zone matrix (GLDZM) based features, as described by Thibault et al. (13). A grey level distance-zone matrix describes the amount of homogeneous connected areas within the volume, of a certain intensity and distance to the shape border. The shape border is defined by 6-connectedness in 3D (i.e. a voxel is on the border, if at least one face is exposed). In contrast to the original definition by Thibault et al. (13), the minimum distance to the border is 1, instead of 0 (i.e. voxels on the border have a distance of 1), to allow for correct feature calculations. The (i,j)th entry of the GLDZM $p(i,j)$ is the number of connected areas of grey level (i.e. intensity value) i and minimum distance j to the

shape border. GLSZM features therefore describe the radial distribution of homogeneous areas within the tumor volume. Let:
$p(i,j)$ be the (i,j)th entry in the given GLDZM p,
N_g the number of discrete intensity values in the image,
N_d the largest distance of a homogeneous region in the volume of interest to the shape border,
N_s the total number of homogeneous regions (zones), where $N_s = \sum_{i=1}^{N_g} \sum_{j=1}^{N_d} p(i,j)$,
p_d the sum distribution of the number of zones with distance j, where $p_z(j) = \sum_{i=1}^{N_g} p(i,j)$,
p_g the sum distribution of the number of zones with grey level i, where $p_g(i) = \sum_{j=1}^{N_z} p(i,j)$,
N_p the number of voxels in the image, where $N_p = \sum_{j=1}^{N_d} jp_d$,
$p_n(i,j)$ the normalized size-zone matrix, where $p_n(i,j) = \frac{p(i,j)}{N_s}$,
μ_d the mean distance, where $\mu_d = \sum_{i=1}^{N_g} \sum_{j=1}^{N_d} jp_n(i,j|\theta)$,
μ_g the mean grey level, where $\mu_g = \sum_{i=1}^{N_g} \sum_{j=1}^{N_d} ip_n(i,j|\theta)$.

1. Small distance Emphasis (SDE)

$$SDE = \frac{1}{N_s} \sum_{j=1}^{N_d} \frac{p_d}{j^2}$$

2. Large distance Emphasis (LDE)

$$LDE = \frac{1}{N_s} \sum_{j=1}^{N_d} j^2 p_d$$

3. Intensity Non-Uniformity (IN)

$$IN = \frac{1}{N_s} \sum_{i=1}^{N_g} p_g^2$$

4. Intensity Non-Uniformity Normalized (INN)

$$INN = \frac{1}{N_s^2} \sum_{i=1}^{N_g} p_g^2$$

5. Distance-zone Non-Uniformity (DZN)

$$DZN = \frac{1}{N_s} \sum_{j=1}^{N_d} p_d^2$$

6. Distance-zone Non-Uniformity Normalized (DZNN)

$$DZNN = \frac{1}{N_s^2} \sum_{j=1}^{N_d} p_d^2$$

7. Zone Percentage (ZP)

$$ZP = \frac{N_s}{N_p}$$

8. Low intensity Emphasis (LIE)

$$LIE = \frac{1}{N_s}\sum_{i=1}^{N_g}\frac{p_g}{i^2}$$

9. High intensity Emphasis (HIE)

$$HIE = \frac{1}{N_s}\sum_{i=1}^{N_g} i^2 p_g$$

10. Low intensity small distance Emphasis (LISDE)

$$LISDE = \frac{1}{N_s}\sum_{i=1}^{N_g}\sum_{j=1}^{N_d}\frac{p(i,j)}{i^2 j^2}$$

11. High intensity small distance Emphasis (HISDE)

$$HISDE = \frac{1}{N_s}\sum_{i=1}^{N_g}\sum_{j=1}^{N_d}\frac{p(i,j)i^2}{j^2}$$

12. Low intensity large distance Emphasis (LILDE)

$$LILDE = \frac{1}{N_s}\sum_{i=1}^{N_g}\sum_{j=1}^{N_d}\frac{p(i,j)j^2}{i^2}$$

13. High intensity large distance Emphasis (HILDE)

$$HILDE = \frac{1}{N_s}\sum_{i=1}^{N_g}\sum_{j=1}^{N_d} p(i,j)i^2 j^2$$

14. Intensity variance (IV)

$$IV = \sum_{i=1}^{N_g}\sum_{j=1}^{N_d}(i-\mu_g)^2 p_n(i,j)$$

15. Distance-zone variance (DZV)

$$SZV = \sum_{i=1}^{N_g}\sum_{j=1}^{N_d}(j-\mu_d)^2 p_n(i,j)$$

16. Distance-zone entropy (DZE)

$$DZE = \sum_{i=1}^{N_g}\sum_{j=1}^{N_d} p_n(i,j)\log_2[p_n(i,j)]$$

Neighborhood Grey tone difference matrix based features

Neighborhood Grey tone difference matrix (NGTDM) based features, as described by Amadasun and King (14). The ith entry of the NGTDM $s(i|d)$ is the sum of grey level differences of voxels with intensity i and the average intensity A_i of their neighboring voxels within a distance d. In contrast to the original paper, a complete neighborhood is not required and A_i is determined over the valid voxels. Let:

n_i be the number of voxels with grey level i,

$N_v = \sum n_i$, the total number of voxels (defined as n^2 by Amadasun and King (14)),

$s(i) = \begin{cases} \sum_{n_i} |i - A_i| & \text{for } n_i > 0 \\ 0 & \text{otherwise} \end{cases}$, generalized for any distance d,

N_g be the maximum discrete intensity level in the image,

$p(i) = \frac{n_i}{N_v}$, the probability of grey level i,

N_p, the total number of grey levels present in the image.

1. Coarseness

$$coarseness = \frac{1}{\varepsilon + \sum_{i=i}^{N_g} p(i)s(i)}$$

Where ε is a small number to prevent coarseness becoming infinite.

2. Contrast

$$contrast = \left(\frac{1}{N_p(1 - N_p)} \sum_{i=1}^{N_g} \sum_{j=1}^{N_g} p(i)p(j)(i - j)^2 \right) \left(\frac{1}{N_v} \sum_{i=i}^{N_g} s(i) \right)$$

3. Busyness

$$busyness = \frac{\sum_{i=i}^{N_g} p(i)s(i)}{\sum_{i=i}^{N_g} \sum_{j=i}^{N_g} |ip(i) - jp(j)|}, \qquad p(i) \neq 0,\ p(j) \neq 0$$

4. Complexity

$$complexity = \frac{1}{N_v} \sum_{i=i}^{N_g} \sum_{j=i}^{N_g} |i - j| \frac{p(i)s(i) + p(j)s(j)}{p(i) + p(j)}, \qquad p(i) \neq 0,\ p(j) \neq 0$$

5. Strength

$$strength = \frac{\sum_{i=i}^{N_g} \sum_{j=i}^{N_g} [p(i) + p(j)](i - j)^2}{\varepsilon + \sum_{i=i}^{N_g} s(i)}, \qquad p(i) \neq 0,\ p(j) \neq 0$$

Neighboring Grey level dependence matrix based features

Neighboring Grey level dependence matrix (NGLDM) based features, as described by Sun and Wee (15). NGLDM features are invariant under spatial rotation. The (i, j)th entry of the NGLDM $p(i, j|d, a)$ describes the number of neighborhoods with center voxel grey level (i.e. intensity value) i and dependence (i.e. number of dependent voxels) $k = j - 1$. A neighborhood are all voxels within a

distance d from the center voxel. The center voxel and a neighboring voxel are dependent if their absolute grey value difference $\leq a$, the dependency coarseness parameter. The features originally specified by Sun and Wee are analogous to the GLRLM and GLSZM features, and the feature set is extended accordingly. Let:

$p(i,j)$ be the (i,j)th entry in the given NGLDM p, generalized for any d and a,

N_g the number of discrete intensity values in the image,

N_d the maximum dependence value,

N_s the total number of neighborhoods, where $N_s = \sum_{i=1}^{N_g} \sum_{j=1}^{N_z} p(i,j)$,

p_d the sum distribution of the number of neighborhoods with dependence $j = k + 1$, where $p_d(j) = \sum_{i=1}^{N_g} p(i,j)$,

p_g the sum distribution of the number of neighborhoods with center voxel grey level i, where $p_g(i) = \sum_{j=1}^{N_d} p(i,j)$,

$p_n(i,j)$ the normalized NGLDM, where $p_n(i,j) = \frac{p(i,j)}{N_s}$,

μ_d the mean dependence, where $\mu_r = \sum_{i=1}^{N_g} \sum_{j=1}^{N_z} j p_n(i,j|\theta)$,

μ_g the mean grey level, where $\mu_g = \sum_{i=1}^{N_g} \sum_{j=1}^{N_z} i p_n(i,j|\theta)$.

Note: By definition, the number of voxels in the image (N_p) equals the total number of neighborhoods (N_s), since in our implementation every voxel is considered to have a neighborhood. Feature "dependence percentage" $\left(\frac{N_s}{N_p}\right)$, which is the equivalent to run-length feature "run percentage" (RP; 7), is therefore omitted, because it will always evaluate to 1.

1. Small Dependence Emphasis (SDE)

$$SDE = \frac{1}{N_s} \sum_{j=1}^{N_d} \frac{p_d}{j^2}$$

This feature is also called Small Number Emphasis (15).

2. Large Dependence Emphasis (LDE)

$$LDE = \frac{1}{N_s} \sum_{j=1}^{N_d} j^2 p_d$$

This feature is also called Large Number Emphasis (15).

3. Grey-level Non-Uniformity (GLN)

$$GLN = \frac{1}{N_s} \sum_{i=1}^{N_g} p_g^2$$

4. Grey-level Non-Uniformity Normalized (GLNN)

$$GLNN = \frac{1}{N_s^2} \sum_{i=1}^{N_g} p_g^2$$

5. Dependence Non-Uniformity (DN)

$$DN = \frac{1}{N_s}\sum_{j=1}^{N_d} p_z^2$$

This feature is also called Number Nonuniformity (15).

6. Dependence Non-Uniformity Normalized (DNN)

$$DNN = \frac{1}{N_s^2}\sum_{j=1}^{N_z} p_z^2$$

7. Low Grey-level Emphasis (LGE)

$$LGE = \frac{1}{N_s}\sum_{i=1}^{N_g} \frac{p_g}{i^2}$$

8. High Grey-level Emphasis (HGE)

$$HGE = \frac{1}{N_s}\sum_{i=1}^{N_g} i^2 p_g$$

9. Low Grey-level small Dependence Emphasis (LGSDE)

$$LGSDE = \frac{1}{N_s}\sum_{i=1}^{N_g}\sum_{j=1}^{N_d} \frac{p(i,j)}{i^2 j^2}$$

10. High Grey-level small Dependence Emphasis (HGSDE)

$$HGSDE = \frac{1}{N_s}\sum_{i=1}^{N_g}\sum_{j=1}^{N_d} \frac{p(i,j) i^2}{j^2}$$

11. Low Grey-level large Dependence Emphasis (LGLDE)

$$LGLDE = \frac{1}{N_s}\sum_{i=1}^{N_g}\sum_{j=1}^{N_d} \frac{p(i,j) j^2}{i^2}$$

12. High Grey-level large Dependence Emphasis (HGLDE)

$$HGLDE = \frac{1}{N_s}\sum_{i=1}^{N_g}\sum_{j=1}^{N_d} p(i,j) i^2 j^2$$

13. Grey-level variance (GLV)

$$GLV = \sum_{i=1}^{N_g}\sum_{j=1}^{N_d} (i - \mu_g)^2 p_n(i,j)$$

14. Dependence variance (DV)

$$DV = \sum_{i=1}^{N_g} \sum_{j=1}^{N_d} (j - \mu_d)^2 p_n(i,j)$$

15. Dependence entropy (DE), also called Entropy (15)

$$DE = \sum_{i=1}^{N_g} \sum_{j=1}^{N_d} p_n(i,j) \log_2 [p_n(i,j)]$$

Note: the definition of entropy by Sun and Wee (15) uses the dependence counts (p) instead of the dependence propabilities (p_n).

16. Second moment (SM) (15)

$$SM = \frac{\sum_{i=1}^{N_g} \sum_{j=1}^{N_d} p(i,j)^2}{N_s}$$

Note: for this feature, defined by Sun and Wee (15), there is no grey-level run-length equivalent

Wavelet features

Wavelet transform effectively decouples textural information by decomposing the original image, in a similar manner as Fourier analysis, in low– and high-frequencies. In this study a discrete, one-level and undecimated three dimensional wavelet transform was applied to each CT and CBCT image, which decomposes the original image X into 8 decompositions. Consider L and H to be a low-pass (i.e. a scaling) and, respectively, a high-pass (i.e. a wavelet) function, and the wavelet decompositions of X to be labeled as X_{LLL}, X_{LLH}, X_{LHL}, X_{LHH}, X_{HLL}, X_{HLH}, X_{HHL} and X_{HHH}. For example, X_{LLH} is then interpreted as the high-pass sub band, resulting from directional filtering of X with a low-pass filter along x-direction, a low pass filter along y-direction and a high-pass filter along z-direction and is constructed as:

$$X_{LLH}(i,j,k) = \sum_{p=1}^{N_L} \sum_{q=1}^{N_L} \sum_{r=1}^{N_H} L(p)L(q)H(r)X(i+p, j+q, k+r)$$

Where N_L is the length of filter L and N_H is the length of filter H. The other decompositions are constructed in a similar manner, applying their respective ordering of low or high-pass filtering in x, y and z-direction. Wavelet decomposition of the image X is schematically depicted in Figure 7. Since the applied wavelet decomposition is undecimated, the size of each decomposition is equal to the original image and each decomposition is shift invariant. Because of these properties, the original tumor delineation of the gross tumor volume (GTV) can be applied directly to the decompositions after wavelet transform. In this study “Coiflet 1” wavelet was applied. For each decomposition we computed the first order gray level statistics and the textural features (GLCM, GLRLM, GLSZM).

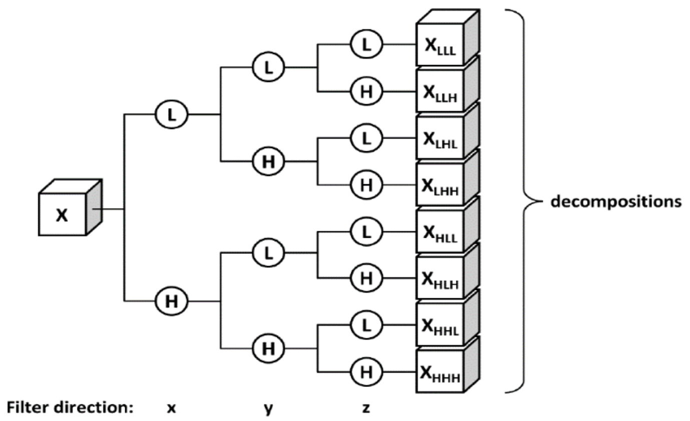

Figure 8: Schematic of the undecimated three dimensional wavelet transform applied to each CT/CBCT image. The original image X is decomposed into 8 decompositions, by directional low-pass (i.e. a scaling) and high-pass (i.e. a wavelet) filtering: X_{LLL}, X_{LLH}, X_{LHL}, X_{LHH}, X_{HLL}, X_{HLH}, X_{HHL} and X_{HHH}.

REFERENCES Supplementary Material

1. Al-Kadi OS, ast, Watson D. Texture Analysis of Aggressive and Nonaggressive Lung Tumor CE CT Images. IEEE Transactions on Biomedical Engineering. 2008;55(7):1822-30.
2. Wahl RL, Jacene H, Kasamon Y, Lodge MA. From RECIST to PERCIST: Evolving Considerations for PET response criteria in solid tumors. Journal of nuclear medicine : official publication, Society of Nuclear Medicine. 2009;50 Suppl 1:122s-50s.
3. Aerts HJ, Velazquez ER, Leijenaar RT, Parmar C, Grossmann P, Carvalho S, et al. Decoding tumour phenotype by noninvasive imaging using a quantitative radiomics approach. Nat Commun. 2014;5:4006.
4. Sousa JR, Silva AC, de Paiva AC, Nunes RA. Methodology for automatic detection of lung nodules in computerized tomography images. Comput Methods Programs Biomed. 2010;98(1):1-14.
5. Haralick RM, Shanmugam K, Dinstein I. Textural Features of Image Classification. IEEE T Syst Man Cyb. 1973;SMC-3(6):610-21.
6. Clausi DA. An analysis of co-occurrence texture statistics as a function of grey level quantization. Canadian Journal of remote sensing. 2002;28(1):45-62.
7. Tsatsoulis LSaC. Texture Analysis of SAR Sea Ice Imagery Using Gray Level Co-Occurrence Matrices. IEEE Transactions on Geoscience and Remote Sensing. 1999;37(2).
8. Galloway M. Texture analysis using gray level run lengths. Comput Vision Graph. 1975;4:172-9.
9. Albregtsen F, Nielsen B, Danielsen HE, editors. Adaptive gray level run length features from class distance matrices. Pattern Recognition, 2000 Proceedings 15th International Conference on; 2000 2000.
10. Thibault GF, B; Navarro, C; Pereira, S. Texture indexes and gray level size zone matrix: application to cell nuclei classification. Pattern Recognition Inf Process. 2009:140-5.
11. Tixier F, Hatt M, Le Rest CC, Le Pogam A, Corcos L, Visvikis D. Reproducibility of tumor uptake heterogeneity characterization through textural feature analysis in 18F-FDG PET. J Nucl Med. 2012;53(5):693-700.
12. Tixier F, Le Rest CC, Hatt M, Albarghach N, Pradier O, Metges JP, et al. Intratumor heterogeneity characterized by textural features on baseline 18F-FDG PET images predicts response to concomitant radiochemotherapy in esophageal cancer. J Nucl Med. 2011;52(3):369-78.
13. Thibault G, Angulo J, Meyer F. Advanced Statistical Matrices for Texture Characterization: Application to Cell Classification. IEEE Transactions on Biomedical Engineering. 2014;61(3):630-7.
14. Amadasun M, King R. Textural features corresponding to textural properties. Systems, Man and Cybernetics, IEEE Transactions on. 1989;19(5):1264-74.
15. Sun C, Wee WG. Neighboring gray level dependence matrix for texture classification. Computer Vision, Graphics, and Image Processing. 1983;23(3):341-52.
